# First Report of Mermithidae (Enoplea: Mermithida) Parasitizing Adult Stable Flies in Japan

**DOI:** 10.2478/jofnem-2024-0022

**Published:** 2024-05-25

**Authors:** Kaori Shimizu, Taizo Saito, Yasuhiro Takashima, Haruhiko Okada, Mitsuhiko Asakawa, Yasuo Inoshima

**Affiliations:** Joint Department of Veterinary Medicine, Gifu University, Gifu, Gifu 501-1193, Japan; Education and Research Center for Food Animal Health, Gifu University (GeFAH), Gifu, Gifu 501-1193, Japan; Joint Graduate School of Veterinary Sciences, Gifu University, Gifu, Gifu 501-1193, Japan; Center for One Medicine Innovative Translation Research (COMIT), Gifu University, Gifu, Gifu 501-1193, Japan; School of Veterinary Medicine, Rakuno Gakuen University, Ebetsu, Hokkaido 069-8501, Japan

**Keywords:** blood-feeding pests, genetics, host–parasite relationship, insect parasitism, Mermithidae, stable fly

## Abstract

Mermithidae is a family of nematodes that parasitize a wide range of invertebrates worldwide. Herein, we report nematodes that were unexpectedly found in three of 486 adult stable flies (*Stomoxys calcitrans*) captured from three farms (F1, F2, and F3) in different regions of Gifu Prefecture, Japan. We aimed to characterize these nematodes both at the morphological and molecular level. Morphological studies revealed that the nematodes were juveniles of Mermithidae. Phylogenetic analysis based on 18S and 28S rDNA indicated that the mermithids from farms F1 and F2 could be categorized into the same cluster as *Ovomermis sinensis* and *Hexamermis* sp., whereas the mermithid from farm F3 clustered with *Amphimermis* sp. Additionally, these mermithids could be categorized within the same clusters as related mermithids detected in Japan that parasitize various arthropod orders. Our findings suggest that these stable flies may have been parasitized by mermithids already present in the region and that genetically distinct species of mermithids occur across Japan. To the best of our knowledge, this is the first report of mermithids parasitizing adult stable flies in Japan.

Mermithid nematodes (Enoplea: Mermithidae) parasitize a wide range of invertebrates (Nickle, 1972; Platzer, 2007; Poinar, 2015), and previous studies have reported several cases of mermithid parasitism in Japan (Supplementary Table S1). Mermithids have gained attention as biological alternatives to chemical insecticides for the control of agricultural pests (Nickle, 1981; Hidaka and Andow, 2017) and disease vectors (Molloy, 1981; Platzer, 1981; Sanad et al., 2017) because of their ability to cause host mortality. However, biological knowledge of most mermithids, including their host preferences, distribution, and parasitism rates, remains limited. Additionally, the species-level classification of mermithids is challenging because of the scarcity of published morphological information and DNA sequence data for Mermithidae.

Stable flies (Diptera: Muscidae: *Stomoxys calcitrans*) are economically significant blood-feeding pests that pose a threat to livestock globally. Their painful bites not only disrupt the grazing behavior of livestock but also cause direct harm through blood loss, tissue damage, and allergic reactions (Taylor et al., 2012; Machtinger et al., 2021). Moreover, stable flies may play a crucial role in the spread of infectious diseases, particularly in livestock, because of their potential as mechanical vectors of various pathogens, including viruses, bacteria, protozoans, and helminths (Baldacchino et al., 2013; Cook, 2020; Rochon et al., 2021).

In this study, we observed nematodes resembling mermithids inside adult stable flies at three farms in different regions of the Gifu Prefecture, Japan. To the best of our knowledge, there has been no report on nematodes in adult stable flies in Japan. To characterize them morphologically and molecularly, microscopic observations and sequence analyses of the 18S and 28S rDNA genes were carried out. Subsequently, the phylogenetic relationships of the species to the available Mermithidae sequences were analyzed based on the rDNA sequences.

## Materials and Methods

### Adult stable fly sampling and nematode collection

**Figure 1.**
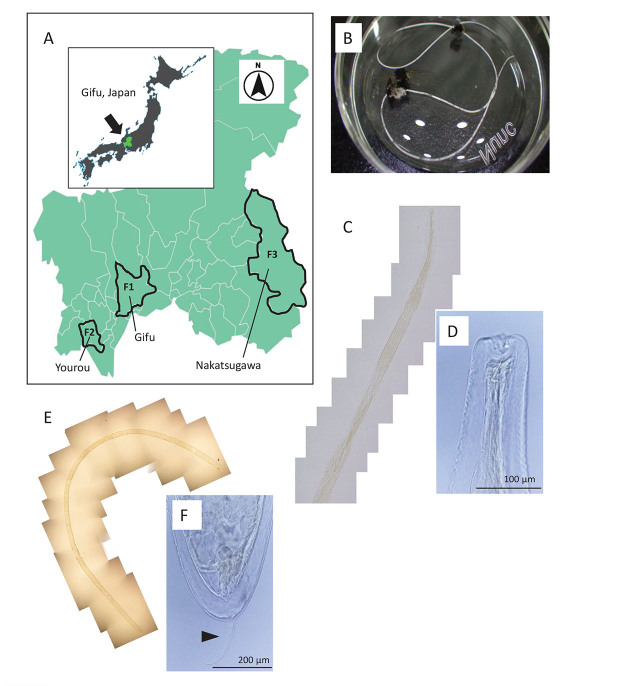
(A) Sampling location of adult stable flies (*Stomoxys calcitrans*). (B—F) Larval form of the mermithid nematode from Gifu. (B) Whole body. (C, D) Anterior part and its extremity. (E, F) Posterior part and its extremity with a tail appendage (arrowhead).

During our study period on surveillance and control measures for flies in farms (Shimizu et al., 2023), adult stable flies were collected from three farms (F1, F2, and F3) across different geographic locations in the Gifu Prefecture, Japan (Table 1 and Figure 1A). Flies were captured once annually from 2021 to 2023 at farms F1 and F2, and once at farm F3 in 2022. Flies inside and outside the livestock barns were captured using butterfly nets. After collection, the flies were transferred to our laboratory and killed by placing them at −80°C. At the time of the use of flies, three nematodes were unexpectedly isolated individually from three flies. The isolated nematodes were fixed in 70% ethanol. Nematodes 1 (Gifu), 2 (Yourou), and 3 (Nakatsugawa) were isolated from stable flies captured from farms F1, F2, and F3, respectively (Table 1).

**Table 1.** Detection of parasitic nematodes from *Stomoxys calcitrans* on three farms in the Gifu Prefecture, Japan.

**Farm**	**Location**	**Sampling date**	**Total stable flies (Number)**	**Parasitized stable flies (Number)**	**Total nematodes (Number)**
Dairy cattle farm					
F1	Gifu	11 Oct 2021	80	1	1^a^
		15 Sep 2022	140	0	0
		20 Sep 2023	112	0	0
		Subtotal	332	1	1
F2	Yourou	8 Oct 2021	17	1	1^b^
		18 Oct 2022	38	0	0
		11 Oct 2023	27	0	0
		Subtotal	82	1	1

Sheep farm					
F3	Nakatsugawa	29 Jul 2022	72	1	1^c^
Total			486	3	3

a Nematode 1 (Gifu).

b Nematode 2 (Yourou).

c Nematode 3 (Nakatsugawa).

### Morphological and genetic analyses

For morphological observation, the specimens were cleared in a glycerol-ethanol solution (5% glycerol in 70% ethanol) by the evaporation of ethanol and were mounted on glass slides with 50% glycerol solution. The specimens were viewed under Nikon Optiphot (Nikon, Tokyo, Japan) and Olympus BX50 (Olympus, Tokyo, Japan) microscopes equipped with a commercial camera Lucida.

A molecular approach was used to identify the nematode species. Total DNA was extracted from the middle part of the nematode body using a DNeasy Blood and Tissue Kit (QIAGEN, Venlo, Netherlands). Several primer sets were used to amplify 18S and 28S rDNA (Supplementary Table S2). PCR was performed with GoTaq Hot Start Green Master Mix (Promega, Madison, WI, USA) using a Veriti thermal cycler (Applied Biosystems, Foster City, CA, USA). The PCR conditions are listed in Supplementary Table S2. The PCR products were purified using NucleoSpin Gel and PCR Clean-up (Macherey-Nagel, Duren, Germany), and the nucleotide sequences were determined via direct sequencing using a BigDye Terminator Cycle Sequencing Kit v3.1 (Applied Biosystems). DNA sequences were confirmed and edited using Genetyx-Win version 13 software (Genetyx, Tokyo, Japan), and the species were identified based on the results of the Basic Local Alignment Search Tool (BLAST) analysis. The obtained sequences were deposited in the DDBJ/EMBL/GenBank database under the following accession numbers: LC788412 to LC788414 for 18S rDNA and LC788415 to LC788417 for 28S rDNA.

The DNA sequences of the mermithids available in the NCBI database are highly variable in length. Therefore, to include reference sequences from known species in the phylogenetic analysis, we aligned 327 to 986 bp for the 18S and 296 to 822 bp for the 28S rDNA sequences. Furthermore, to include reference sequences with known hosts in the phylogenetic analysis, we aligned 327 to 1011 bp of the 18S rDNA sequences. Phylogenetic trees were constructed using the maximum-likelihood method with the Kimura two-parameter model, and the reliability of the branches was evaluated using 1,000 replicates. In total, 76 sequences of previously reported mermithid nematodes from Japan and other countries were used for the phylogenetic analysis (Supplementary Table S3).

## Results

Three nematodes were found individually in three of the 486 stable flies from three farms in Gifu, Japan (Table 1). At each of the farms (F1, F2, and F3), only a single adult stable fly of the 332, 82, and 72 flies, respectively, sampled was parasitized by nematodes, indicating a low prevalence of the nematode. Previous studies have reported negative effects on hosts infected with mermithids, including deformation (Muñoz-Muñoz et al., 2016; Mazza et al., 2017). However, differences in the general appearance were not observed between the three infected flies and the other flies.

### Morphological characterization

The morphological characteristics of the mermithids are shown in Figures 1B—1F. The nematodes were wire-like in shape and had a milky-white cuticle sheath. The specimen from farm F1 was ca. 12.5 cm in length and ca. 0.25 mm in width. Among the nematodes, the sample from farm F1 (Figure 1A) could be studied morphologically, as it had relatively complete head and tail extremities (Figures 1C–1F). The head had a slender esophagus, and the tail had an appendage on the posterior end. Thus, this nematode was a juvenile belonging to the family Mermithidae. Based on the general appearance and host range, the other two degenerated individuals from farms F2 and F3 could belong to the same family and stage (Poinar, 1975). This is further supported by the sequence analysis described in the following sections.

### Molecular characterization

The lengths of the three 18S rDNA sequences were as follows: Gifu, 1,662 bp; Yourou, 1,733 bp; and Nakatsugawa, 1,719 bp. BLAST analysis showed that nematodes from Gifu and Youro shared 99.94% (1603/1604) and 99.82% (1633/1636) identity, respectively, with *Hexamermis* sp. (LC661691) isolated from *Glaucias subpunctatus* (Hemiptera) in Mie, Japan (Watanabe et al., 2020). In addition, the Nakatsugawa specimen shared 99.21% (630/635) identity with *Amphimermis enzoni* (MT021436) isolated from *Ischnura fluviatilis* and *Rhionaeschna bonariensis* (Odonata) in Argentina (Rusconi et al, 2020) and 99.10% (774/781) with *Amphimermis* sp. (EF617354) isolated in China. The lengths of the three 28S rDNA sequences were as follows: Gifu, 1,103 bp; Yourou, 1,103 bp; and Nakatsugawa, 1,082 bp. BLAST analysis showed that Gifu and Yourou exhibited 90.39% (715/791) and 90.52% (716/791) identity, respectively, with *Hexamermis agrotis* (KC784667) isolated in Turkey. In addition, Nakatsugawa shared 96.18% sequence identity with *Amphimermis* sp. (EF617372) isolated in China.

**Figure 2.**
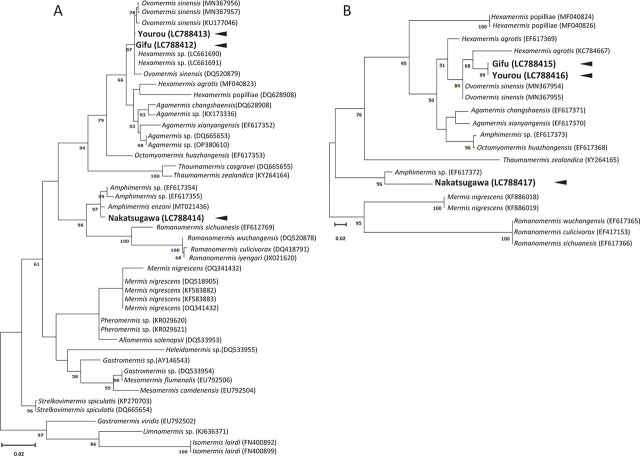
Phylogenetic trees of Mermithidae with known genus-level identifications based on 18S (A) and 28S (B) rDNA using the maximum-likelihood method. Arrowheads indicate the mermithids sequenced in this study. Bootstrap values above 50% are indicated at the phylogenetic tree node.

Phylogenetic trees based on the alignment of 18S and 28S rDNA sequences classified the mermithids from Gifu and Yourou into clusters containing *Ovomermis sinensis* reported in China (MN3679544 to MN367957, and DQ520879) and Poland (KU177046), and *Hexamermis* sp. (LC661690 and LC661691) reported in Japan, whereas the specimen from Nakatsugawa fell into a cluster containing *Amphimermis* sp. reported in Argentina (MT021436) and China (EF617354, EF617355, and EF617372) (Figure 2).

**Figure 3.**
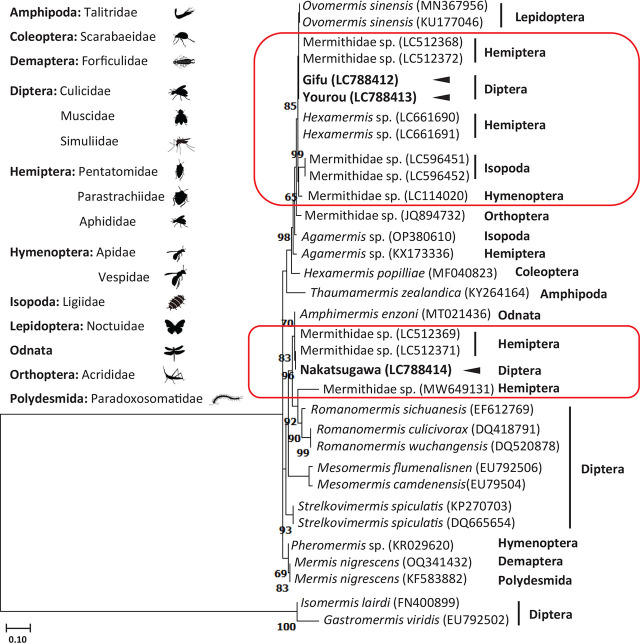
Phylogenetic tree of Mermithidae with known hosts based on 18S rDNA sequences using the maximum-likelihood method. Arrowheads indicate the mermithids sequenced in this study. The red frame includes mermithids recorded from Japan. Bootstrap values above 65% are indicated at the phylogenetic tree node.

**Figure 4.**
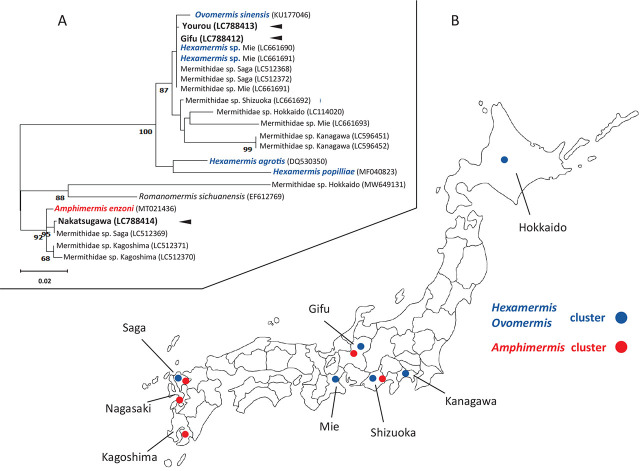
Phylogenetic tree of unidentified species of mermithid nematodes parasitizing insects in Japan based on 18S rDNA sequences (A) using the maximum-likelihood method and (B) distribution map of mermithids included in *Ovomermis sinensis, Hexamermis* sp., and *Amphimermis* sp. clusters. *Ovomermis sinensis* (KU177046), *Hexamermis agrotis* (DQ530350), *Hexamermis popilliae* (MF040823), *Romanomerimis sichuanensis* (EF612769), and *Amphimermis enzoni* (MT021436) have been identified in other countries and used as reference species in the phylogenetic tree. Arrowheads indicate the mermithids isolated in Gifu and sequenced in this study. Bootstrap values above 65% are indicated at the phylogenetic tree node.

A phylogenetic tree, constructed using 18S rDNA sequences and referencing known host information, revealed that specimens from Gifu and Yourou formed a distinct cluster encompassing hosts such as Isopoda, Hemiptera, Hymenoptera, and Lepidoptera (Figure 3). In contrast, the Nakatsugawa specimen was positioned within a cluster that included Hemiptera and Odonata hosts. Additionally, these mermithids could be categorized into the same clusters as other mermithids recorded from Japan, rather than in clusters within which the host species were categorized (Figure 3). Phylogenetic analysis of 13 unidentified species of mermithids that parasitize insects in Japan (registered in GenBank) categorized the three mermithids we sampled into clusters with *O. sinensis*, *Hexamermis* sp., or *Amphimermis* sp., with one exception (MW649131 isolated from *Erisoma auratum* in Hokkaido; Figure 4, Supplementary Table S4).

## Discussion

This is the first report of mermithids isolated from adult stable flies in Japan. This study identified mermithid parasitization in merely three out of 486 flies. Notably, the occurrence of mermithid parasitism in adult stable flies has been previously reported only once, originating from cattle farms and suburbs in the USA (Smith et al., 1987). These results suggest that mermithid parasitism is extremely rare in adult stable flies.

Morphological identification of mermithids must be performed using adults, which constitute the free stage of these parasites, as the genitalic structures and other organs necessary for identification are fully formed specifically in this stage (Poinar, 1979). However, the Mermithidae nematodes obtained in this study were in the larval form; therefore, accurate identification could not be performed. Species identification based on morphological observation remains unclear, whereas sequence analysis is a well-developed powerful tool for genetic identification (St-Onge et al., 2008; Yoshino et al., 2021). Molecular phylogenetic analysis revealed that the mermithids parasitizing adult stable flies formed two different clusters with *O. sinensis* and *Hexamermis* sp. or *Amphimermis* sp. and that these clusters included hosts of various orders. The high sequence identities of the stable fly mermithids with these aforementioned species suggest that they may belong to these or closely related genera.

In terrestrial mermithids, including *Hexamermis* sp. and *Amphimermis* sp., infective juveniles seek out a host and bore through the host’s body wall into the body cavity (Nguyen, 2011). Additionally, *Hexamermis* sp. (Achinelly and Camino, 2008) and *Amphimermis* sp. (Baker and Poinar, 1994) parasitism has been reported in Coleoptera, Dermaptera, Diptera, Hemiptera, Homoptera, Hymenoptera, Lepidoptera, Odonata, and Orthoptera. Therefore, stable flies may also be viable hosts for these terrestrial mermithids.

Mermithids that parasitize adult stable flies tended to be classified into the same clusters as mermithids previously recorded in Japan, and most unidentified species of mermithids that parasitize insects in Japan were classified into clusters with *O. sinensis* and *Hexamermis* sp. or *Amphimermis* sp. (Figure 4). Our results, along with those of previous reports (Supplementary Table S1), confirm that *O. sinensis*, *Hexamermis* sp., and *Amphimermis* sp. are distributed in Japan.

## Figures and Tables

**Supplementary Table S1.** Mermithid nematodes reported in Japan.

**Species**	**Location**	**Host**	**Reference**
*Agamermis unka*	Hiroshima	*Sogatella furcifera Nilaparvata lugens* (Homoptera: Delphacidae)	Hidaka and Andow, 2017
*Amphimermis zuimushi*	Shizuoka	*Chilo simplex* Butler (Lepidoptera: Pyralidae)	Kaburaki and Imamura, 1932
*Gastromermis mesostoma lsomermis bipapillatus*	Fukuoka, Kagoshima, Oita	*Simulium japonicum* (Diptera: Simuliidae)	Poinar et al., 1986
*Hexamermis* sp. *Mermithidae* sp.	Mie, Saga, Shizuoka	*Glaucias subpunctatus Plautia stali* (Hemiptera: Parastrachiidae, Pentatomidae)	Watanabe et al., 2021
Mermithidae sp.	Hokkaido	*Bombus pseudobaicalensis* Vogt (Hymenoptera: Bombinae)	Kubo et al., 2016
Mermithidae sp.	Kanagawa	*Ligidium* sp. (Isopoda: Ligiidae)	Yoshino and Waki, 2021
Mermithidae sp.	Hokkaido	*Eriosoma auratum* (Hemiptera: Aphididae)	Tong et al., 2021
Mermithidae sp.	Saga, Nagasaki, Kagoshima	*Parastrachia japonensis Plautia stali* (Hemiptera: Parastrachiidae, Pentatomidae)	Iryu et al., 2020
Mermithidae sp.	Kyoto	*Pardosa pseudoannulata* (Araneae: Lycosidae)	Iida et al., 2003

**Supplementary Table S2.** Primers and PCR conditions used in this study.

**Target**	**Primers**	**Sequence (5′-3′)**	**PCR conditions**				**Reference**
			**Denaturation**	**Annealing**	**Extension**	**Cycles**	
18S rDNA	MF	CAAGGACGAAAGTTAGAGGTTC	95°C, 30sec	50°C, 30sec	72°C, 45sec	40	Kobylinski et al. 2012
	MR	GGAAACCTTGTTACGACTTTTA					
	KuboF	ATGCATGTCTAAGCACATGCC	95°C, 30sec	50°C, 1min	72°C, 2min	40	Kubo et al. 2016
	KuboR	AACCTTGTTACGACTTTTACTTC					
	newF	AGTTATCTACTTGGATAACTGTG					This study
28S rDNA	F (3745-3764)	AGCGGAGGAAAAGAAACTAA	95°C, 30sec	62°C, 30sec	72°C, 1min	35	Nadler et al. 1998
	R (4359-4377)	ATCCGTGTTTCAAGACGGG					
	LSU-F	ACAAGTACCGTGAGGGAAAGTTG	94°C, 30sec	45°C, 1min	72°C, 2min	40	Tong et al. 2021
	LSU-R	TCGGAAGGAACCAGCTACTA					

**Supplementary Table S3.** Mermithid nematodes used in phylogenetic analysis of 18S and 28S rDNA retrieved from GenBank^a^.

**Organism**	**Location**	**Host**	**Accession number**		**Reference**
			**18S rDNA**	**28S rDNA**	
*Agamermis changshaensis*	-	-	DQ628908	-	Unpublished
*Agamermis changshaensis*	-	-	-	EF617371	Unpublished
*Agamermis xianyangensis*	-	-	EF617352	EF617370	Unpublished
*Agamermis* sp.	Argentina United States	*Armadillidium vulgare* (Isopoda: Armadillidiidae)	OP380610	-	Unpublished
		*Megacopta cribraria* (Hemiptera: Plataspidae)	KX173336	-	Stubbins et al. 2016
	-	Environment	DQ665653	-	Unpublished
*Allomermis solenopsii*	-	-	DQ533953	-	Unpublished
*Amphimermis enzoni*	Argentina	*Ischnura fluviatilis Rhionaeschna bonaerensis* (Odnata)	MT021436	-	Rusconi et al. 2020
*Amphimermis* sp.	China	-	EF617354	EF617372	Unpublished
		-	EF617355	EF617373	
*Gastromermis* sp.	-	-	DQ533954	-	Unpublished
	-	-	AY146543	-	Unpublished
*Gastromermis viridis*	Canada	*Simulium squamosum* (Diptera: Simuliidae)	EU792502	-	St-Onge et al. 2008
*Heleidomermis* sp.	-	-	DQ533955	-	Unpublished
*Hexamermis agrotis*	-	-	DQ530350	-	Unpublished
	-	-	-	EF617369	Unpublished
	Turkey	-	-	KC784667	Unpublished
*Hexamermis popilliae*	Italy	*Popillia japonica* (Coleoptera: Scarabaeidae)	MF040823	MF040824	Mazza et al. 2017
			-	MF040826	
*Hexamermis* sp.	Japan	*Glaucias subpunctatus* (Hemiptera: Pentatomidae)	LC661690	-	Watanabe et al. 2021
			LC661691	-	
*Isomermis lairdi*	Ghana	*Simulium squamosum* (Diptera: Simuliidae)	FN400899	-	Crainey et al. 2009
			FN400892	-	
*Limnomermis* sp.	-	-	KJ636371	-	Unpublished
*Mermis nigrescens*	Viet Nam	*Orthomorpha coarctata* (Polydesmida: Paradoxosomatidae)	OQ341432	-	Unpublished
	New	*Forficula auricularia* (Dermaptera: Forficulidae)	KF583882	KF886019	Unpublished
	Zealand	Plant	KF583883	KF886018	
	-	-	AF036641	-	Blaxter et al. 1998
Mermithidae sp.	Japan	*Parastrachia japonensis* (Hemiptera: Parastrachiidae)	LC512368	-	Iryu et al. 2020
			LC512369	-	
			LC512370	-	
			LC512371	-	
	Japan	*Plautia stali* (Hemiptera: Parastrachiidae)	LC512372	-	Iryu et al. 2020
	Japan	*Ligidium* sp. (Isopoda: Ligiidae)	LC596451	LC500234	Yoshino and Waki, 2021
			LC596452	LC500235	
	Japan	*Eriosoma auratum* (Hemiptera: Aphididae)	MW649131	MW653323	Tong et al. 2021
	Japan	*Bombus pseudobaicalensis* (Hymenoptera: Apidae)	LC114020	-	Kubo et al. 2016
	Japan	*Glaucias subpunctatus* (Hemiptera: Pentatomidae)	LC661692	-	Watanabe et al. 2021
	Japan	*Plautia stali (Hemiptera: Parastrachiidae)*	LC661693	-	Watanabe et al. 2021
	Japan	*Stomoxys calcitrans* (Diptera: Muscidae)	LC788412	LC788415	This study
			LC788413	LC788416	
			LC788414	LC788417	
	Australia	*Kosciscola tristis* (Orthoptera: Acrididae)	JQ894731	-	Umbers et al. 2015
			JQ894732	-	
*Mesomermis camdenensis*	Canada	*Simulium squamosum* (Diptera: Simuliidae)	EU792504	-	St-Onge et al. 2008
*Mesomermis flumenalis*	Canada	*Simulium squamosum* (Diptera: Simuliidae)	EU792506	-	St-Onge et al. 2008
*Octomyomermis huazhongensis*	-	-	EF617353	EF617368	Unpublished
*Ovomermis sinensis*	China	*Spodoptera frugiperda* (Lepidoptera: Noctuidae)	MN367957	MN367955	Sun et al. 2020
			MN367956	MN367954	
	Poland	*Cerapteryx graminis* (Lepidoptera: Noctuidae)	KU177046	-	Unpublished
	-	-	DQ520879	-	Unpublished
*Pheromermis* sp.	France	*Vespa velutina* (Hymenoptera: Vespidae)	KR029620	KR029622	Villemant et al. 2015
			KR029621	KR029623	
*Romanomermis culicivorax*	-	*Culex* sp. (Diptera: Culicidae)	DQ418791	-	Unpublished
	-	-	-	EF417153	Sonnenberg et al. 2007
*Romanomermis iyengari*	-	-	JX021620	-	Unpublished
*Romanomermis sichuanensis*	-	*Culex* sp. (Diptera: Culicidae)	EF612769	EF617366	Unpublished
*Romanomermis wuchangensis*	-	*Culex quinquefasciatus* (Diptera: Culicidae)	DQ520878	EF617365	Unpublished
*Strelkovimermis spiculatus*	Argentina	*Aedes albifasciatus* (Diptera: Culicidae)	KP270703	-	Belaich et al. 2015
	-	*Aedes* sp. (Diptera: Culicidae)	DQ665654	-	Unpublished
*Thaumamermis cosgrovei*	-	-	DQ665655	-	Unpublished
*Thaumamermis zealandica*	New Zealand	*Bellorchestia quoyana* (Amphipoda: Talitridae)	KY264164	KY264165	Unpublished

a Hyphen represents that the data was not found on the database.

**Supplementary Table S4.** Information on locations of nematode species and hosts corresponding to nematodes used in Figure 4^a^.

Species	Accession number	Location in Japan	Host			Reference
		(Country)	Family	Genus	Species	
*Ovomermis / Hexamermis* cluster						
*Ovomermis sinensis*	KU177046	(Poland)	Noctuidae	*Cerapteryx*	*C. graminis*	Unpublished
*Hexamermis* sp.	LC661690	Mie	Pentatomidae	*Glaucias*	*G. subpunctatus*	Watanabe et al., 2021
	LC661691	Mie	Pentatomidae	*Glaucias*	*G. subpunctatus*	Watanabe et al., 2021
Mermithidae sp.	LC788413	Gifu	Muscidae	*Stomoxys*	*S. calcitrans*	This study
	LC788412	Gifu	Muscidae	*Stomoxys*	*S. calcitrans*	This study
	LC512368	Saga	Parastrachiidae	*Parastrachia*	*P. japonensis*	Iryu et al., 2020
	LC512372	Saga	Parastrachiidae	*Parastrachia*	*P. japonensis*	Iryu et al., 2020
	LC661692	Shizuoka	Pentatomidae	*Glaucias*	*G. subpunctatus*	Watanabe et al., 2021
	LC114020	Hokkaido	Apidae	*Bombus*	*B. pesudobaicalensis*	Kubo et al., 2016
	LC661693	Mie	Pentatomidae	*Plautia*	*P. stali*	Watanabe et al., 2021
	LC596451	Kanagawa	Ligiidae	*Ligidium*	sp.	Yoshino and Waki, 2021
	LC596452	Kanagawa	Ligiidae	*Ligidium*	sp.	Yoshino and Waki, 2021
*Hexamermis agrotis*	DQ530350	(China)	-	-	-	Unpublished
*Hexamermis popilliae*	MF040823	(Italy)	Scarabaeidae	*Popillia*	*P. japonica*	Mazza et al., 2017

*Amphimermis* cluster						
*Amphimermis enzoni*	MT021436	(Argentina)	Coenagrionidae	*Ischnura*	*I. fluviatilis*	Rusconi et al., 2020
			Aeshnidae	*Rhionaeschna*	*R. bonariensis*	
Mermithidae sp.	LC788414	Gifu	Muscidae	*Stomoxys*	*S. calcitrans*	This study
	LC512369	Saga	Parastrachiidae	*Parastrachia*	*P. japonensis*	Iryu et al., 2020
	LC512371	Kagoshima	Parastrachiidae	*Parastrachia*	*P. japonensis*	Iryu et al., 2020
	LC512370	Kagoshima	Parastrachiidae	*Parastrachia*	*P. japonensis*	Iryu et al., 2020

Others						
Mermithidae sp.	MW649131	Hokkaido	Aphididae	*Eriosoma*	*E. auratum*	Tong et al., 2021
*Romanomermis sichuanensis*	EF612769	(China)	-	-	-	Unpublished

a Hyphen represents that the data was not found in the database.
